# Identification of Heat Shock Transcription Factor Genes Involved in Thermotolerance of Octoploid Cultivated Strawberry

**DOI:** 10.3390/ijms17122130

**Published:** 2016-12-17

**Authors:** Wan-Yu Liao, Lee-Fong Lin, Jing-Lian Jheng, Chun-Chung Wang, Jui-Hung Yang, Ming-Lun Chou

**Affiliations:** 1 Institute of Medical Sciences, Tzu-Chi University, Hualien 97004, Taiwan; 98751101@gms.tcu.edu.tw; 2 Department of Life Sciences, Tzu-Chi University, Hualien 97004, Taiwan; leelin@gms.tcu.edu.tw; 3 Department of Molecular Biology and Human Genetics, Tzu-Chi University, Hualien 97004, Taiwan; pudding30167@yahoo.com.tw; 4 Institute of Molecular Medicine, National Tsing-Hua University, Hsinchu 30013, Taiwan; aricwang@itri.org.tw; 5 Biomedical Technology and Device Research Laboratories, Industrial Technology Research Institute, Hsinchu 30011, Taiwan; JHYen@itri.org.tw

**Keywords:** octoploid cultivated strawberry (*Fragaria × ananassa* Duch. cv. Toyonoka), heat shock transcription factor, heat stress, Illumina sequencing, transcriptome, thermotolerance

## Abstract

Heat shock transcription factors (HSFs) are mainly involved in the activation of genes in response to heat stress as well as other abiotic and biotic stresses. The growth, development, reproduction, and yield of strawberry are strongly limited by extreme temperatures and droughts. In this study, we used Illumina sequencing and obtained transcriptome data set from *Fragaria × ananassa* Duchessne cv. Toyonoka. Six contigs and three unigenes were confirmed to encode HSF proteins (FaTHSFs). Subsequently, we characterized the biological functions of two particularly selected unigenes, *FaTHSFA2a* and *FaTHSFB1a*, which were classified into class A2 and B HSFs, respectively. Expression assays revealed that *FaTHSFA2a* and *FaTHSFB1a* expression was induced by heat shock and correlated well with elevated ambient temperatures. Overexpression of *FaTHSFA2a* and *FaTHSFB1a* resulted in the activation of their downstream stress-associated genes, and notably enhanced the thermotolerance of transgenic *Arabidopsis* plants. Besides, both FaTHSFA2a and FaTHSFB1a fusion proteins localized in the nucleus, indicating their similar subcellular distributions as transcription factors. Our yeast one-hybrid assay suggested that FaTHSFA2a has trans-activation activity, whereas FaTHSFB1a expresses trans-repression function. Altogether, our annotated transcriptome sequences provide a beneficial resource for identifying most genes expressed in octoploid strawberry. Furthermore, HSF studies revealed the possible insights into the molecular mechanisms of thermotolerance, thus rendering valuable molecular breeding to improve the tolerance of strawberry in response to high-temperature stress.

## 1. Introduction

High temperature is one of the most crucial abiotic stresses in fields worldwide because it can considerably affect plant growth and crop production [1,2]. When the temperature increases beyond the optimal growth condition, it causes heat-stress responses in higher plants, leads to the inhibition of photosynthesis, and results in chlorophyll degradation [3]. Because of previous exposure to high temperatures, plants develop a series of defense mechanisms to acquire thermotolerance against unavoidable high temperatures, which includes markedly increased transcript levels of heat-stress-responsive genes encoding heat-shock proteins (HSPs) and small HSPs, which function as molecular chaperones for protein quality control under heat stress [4,5]. In addition to HSPs, other regulatory proteins are involved in heat-stress responses, such as dehydration-responsive element-binding transcription factor 2 (DREB2), galactinol synthase 1 (GolS1), and ascorbate peroxidase 2 (APX2) that function to facilitate plant survival under stressful conditions [6,7,8,9].

Heat shock transcription factors (HSFs), the central regulators of heat shock stress response, regulate the expression of many heat-stress-inducible genes by recognizing the conserved binding motifs (heat stress element, HSE) that exist in their promoters in all eukaryotic organisms [10,11]. The HSF family, similar to many transcription factors, has a conserved modular structure with a N-terminal DNA-binding domain (DBD) characterized by a central helix-turn-helix motif; a hydrophobic coiled-coil region (HR-A/B) composed of hydrophobic heptad repeats essential for oligomerization; short peptide motifs required for nuclear import (nuclear localization signals, NLS) and export; and a C-terminal activation domain (CTAD) rich in aromatic, hydrophobic, and acidic amino acids, the so-called AHA motifs [12,13,14,15,16,17]. HSFs utilize their oligomerization domains to form trimers and function as sequence-specific trimeric DNA-binding proteins via the signal transduction pathway to activate the expression of the *HSP* genes [18].

Only a few *HSF* genes were identified in yeast, fruit fly and vertebrates, whereas genomes of Arabidopsis, rice, tomato, and soybean have been reported to contain 21, 25, 18 and 34 *HSF* genes, respectively [11,14,19,20,21,22]. According to structural characteristics and phylogenetic comparisons, HSFs of those plants have been categorized into three major classes (classes A, B, and C), which revealed the difference in their flexible linkers between the A and B parts of the HR-A/B regions [21,23]. Most class A HSFs contain the AHA motifs and activate the transcription of HSPs through trans-activation by binding some basic transcription protein complexes, whereas class B and C HSFs exhibit no trans-activation activity because of the lack of the AHA motif and function as repressors or co-activators [16,21,24]. It has been recently reported that the structure of class B HSFs (except HSFB5) comprises a characteristic tetrapeptide (LFGV) in the C-terminal domain, acting as a repressor domain (RD) [25,26,27]. In addition to their role in heat stress, previous studies have reported that HSFs may play vital roles in plants under abiotic stress conditions: for instance, cold, salt, drought, and oxidative conditions [28,29,30]. Thus, the multiplicity of plant HSFs suggests their functional diversity and complexity in plants.

Recent studies have identified a high number of plant *HSF* genes from more than 20 plant species, including monocots and eudicots, on the basis of the latest development of next-generation sequencing (NGS) technology and availability of the growing number of complete plant genomic and transcriptome sequences resources. Furthermore, 15–56 HSF members were found in any given plant species, including 25 HSF-encoding genes in rice [17,31], 21 in Arabidopsis [17], 30 in maize [17], 24 in tomato [17,27], 25 in pepper [32], 29 in Chinese white pear [33], 17 in Chinese plum [33], 33 in European pear [33], 17 in peach [33], 52 in soybean [17], and at least 56 in wheat [34]. A diploid woodland strawberry species, *Fragaria vesca* Coville, whose haploid genome size was approximately 240 Mb, is the smallest among the rosaceous species [35]. Its complete genome sequence resources have been available since 2010 [36] and referenced to its octoploid cultivated relatives. Furthermore, another well-known-octoploid strawberry cultivar, “Strawberry Festival”, was subjected to transcriptome analysis by using the Roche-454 GS-FLX system. To increase the transcript diversity and discovery in their analysis, Folta et al. [37] examined whole plants, and their detached tissues were subjected to at least one of the 30 specific treatments, including various growth regulators, light conditions, pharmacological agents, or stresses. The cDNA pools isolated from various tissues were sequenced using the Roche-454 GS-FLX system and assembled into more than 32,000 contigs [37]. Comparative genetic mapping analysis of genomes of diploid and octoploid strawberries showed that they shared a high level of macrosynteny and collinearity [38]. Because of its small herbaceous stature, ease of propagation, short reproductive cycle, efficient regeneration and facile transformation, and available genomic and transcriptome resources, strawberry emerges as an advantageous and powerful functional genomics system for testing gene functions and representing many plants of the Rosaceae family [35,36,39,40,41,42].

Garden strawberry (pineapple strawberry), *F. × ananassa* Duchessne, is a hybrid cultivar of two wild *Fragaria* species (*F. virginiana* and *F. chiloensis*). It is a perennial herbaceous plant of the Rosaceae family, has gradually become a crucial and valuable fruit crop for many regional economies worldwide, and offers high levels of healthful compounds. In Taiwan, the most well-known and major cultivated strawberry in fields is *F. × ananassa* Duch. cv. Toyonoka because of its widely appreciated characteristic aroma, bright red color, juicy texture, and sweetness. Genomically, *F. × ananassa* is the most complex form among fruit tree crops and ornamental plants, harboring eight sets of chromosomes (2*n* = 8× = 56) originally derived from as many as four diploid ancestors [39]. In this study, we performed large-scale transcriptome sequencing of *F. × ananassa* Duch. cv. Toyonoka through high-throughput RNA-sequencing and described the sequences of more than 65,000 generated non-redundant (Nr) transcribed unigenes from six tissues. These unigenes were functionally annotated and analyzed considering their gene ontology (GO) and relationship to metabolic pathways. These unigene data sets from our studies will provide researchers with useful foundation for researching gene expression, genomics and functional genomics of not only *F. × ananassa* but also its closely related species.

In this study, we identified nine *HSF* genes with full-length cDNA sequences (*F. × ananassa* Duch. cv. Toyonoka Heat Shock Transcription Factor Protein, *FaTHSF*), determined from 31 contigs and 15 unigenes annotated as HSF proteins according to our results of *de novo* transcriptome sequencing. Two *FaTHSF* genes, *FaTHSFA2a* and *FaTHSFB1a*, were particularly selected not only because they represent class A2 and B1 HSFs, respectively, by multiple alignment and phylogenetic analysis, but also previous studies have revealed their crucial activities as strong heat-associated HSF proteins [43,44,45,46,47,48]. Reverse transcription polymerase chain reaction (RT-PCR) results showed that *FaTHSFA2a* and *FaTHSFB1a* transcripts were induced by high temperature, and their induced expression patterns correlated well with elevated ambient temperatures. We also found that the overexpression of FaTHSFA2a and FaTHSFB1a proteins caused the activation of downstream stress-associated genes, namely *Hsp101*, *MBF1C* (*multiprotein bridging factor 1C*), and *ELIP* (*early light-induced protein 1*), under normal conditions, thus enhancing the thermotolerance of transgenic Arabidopsis. Data from a subcellular localization assay revealed green fluorescent protein (GFP)-fused FaTHSFA2a and FaTHSFB1a proteins situated in the nucleus, suggesting their putative function as transcription factors. In addition, a yeast one-hybrid assay revealed that *FaTHSFA2a*-encoded proteins can be involved in transcriptional activation, whereas FaTHSFB1a functions as a transcriptional repressor. Altogether, our results provide vital information for the further investigation of strawberry as well as its future molecular breeding.

## 2. Results and Discussion

### 2.1. Illumina Sequencing, De Novo Assembly, and Functional Annotation of the Transcriptome

**Figure 1:**
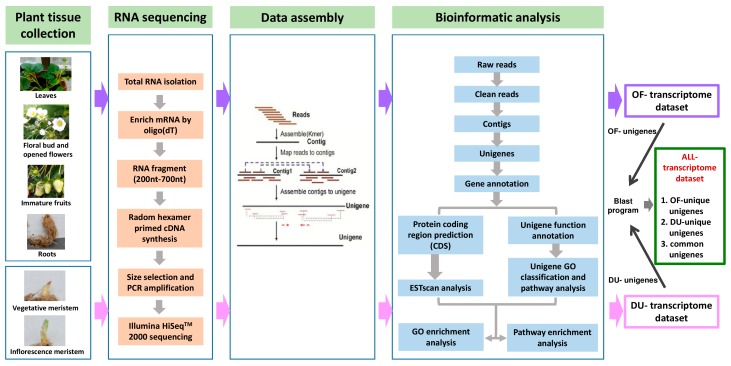
Flowchart of the transcriptome sequencing analysis (plant tissue collection, RNA sequencing, data assembly, and bioinformatics analysis). The total RNA was extracted from five strawberry tissues, namely leaves, floral buds, fully bloomed flowers, immature fruits, and roots. Following cDNA preparation of the extracted RNA and sequencing analysis, the retrieved data were used to set up the OF transcriptome data set. Simultaneously, the total RNA extracted from two other tissues comprising vegetative and inflorescence meristems was utilized to establish the DU transcriptome data set. Sequences of the unigenes obtained either from the OF or DU transcriptome data set were further analyzed, and common genes and unigenes unique to either OF or DU transcriptome data sets (OF- and DU-unique unigenes) were determined. To specify common unigenes, the sequence identity between the two unigenes identified separately from the data sets must be greater than 50%. In addition, its matched length must be higher than 50% of the length of both unigenes. The remaining unigenes appeared as unique to each data set. GO: gene ontology; CDS: consensus coding sequences.

To obtain an overview of the strawberry transcriptome and gene activity at the nucleotide resolution level, we first extracted the total RNA from five strawberry tissues, namely the leaves, floral buds, fully bloomed flowers, immature fruits, and roots, for setting up the OF transcriptome data set. The second preparation involved extracting the total RNA from two distinct tissues containing vegetative and inflorescence meristerms to set up the DU transcriptome data set. Equal amounts of total RNA from each sample were pooled, the mRNA was isolated, enriched, sheared into smaller fragments, and further reverse-transcribed into cDNA. The separated cDNA pools from the two preparations were subjected to Illumina HiSeq^TM^ 2000 sequencing, and the resulting data were examined using bioinformatics analysis. The flowchart of our transcriptome analysis is shown in Figure 1.

After removing adaptor sequences, ambiguous reads, and low-quality reads, we obtained two transcriptome data sets. The data sets contain a total of 4,279,638,060 (4.28 Gb) and 4,412,888,820 (4.41 Gb) nucleotides with high-quality clean reads for OF and DU transcriptomes, respectively (Table 1). All high-quality reads were assembled de novo by using the Trinity program [49]. Consequently, 117,813 contigs, with an N50 of 740 bp representing 50% of the assembled bases were incorporated into contigs of 740 bp or longer in the OF transcriptome data set, and 122,261 contigs, with an N50 of 805 bp in the DU transcriptome data set. An overview of the sequencing and assembly data is outlined in Table 2.

Overview of the size distribution of contigs, unigenes, consensus coding sequences (CDS), and expressed sequence tags (ESTs) from OF, DU, and ALL transcriptome data sets was shown in Figure S1. Briefly, the mean contig size in the OF transcriptome data set was 352 bp (Table 2, Figure S1A). By contrast, the mean contig size in the DU transcriptome data set was 356 bp (Table 2, Figure S1B). In the OF transcriptome data set, a total of 65,164 unigenes were assembled, with an average unigene length of 705 bp and N50 of 1218 bp (Table 2, Figure S1C). Alternatively, a total of 72,914 unigenes were assembled in the DU transcriptome data set. Similarly, the average unigene length was 688 bp with an N50 of 1155 bp (Table 2, Figure S1D). An additional analysis of these unigenes from the two transcriptome data sets revealed common unigenes as well as unigenes unique to each data set. Following integration assays of these two data sets, the ALL transcriptome data set was generated and named. A total of 65,768 unigenes were identified in this data set after combining data from the OF and DU transcriptome data sets (Table 2). These unigenes were with an average length of 873 bp and N50 of 1387 bp (Table 2, Figure S1E). The distribution pattern of unigenes and contigs is similar, except that the unigene size is typically longer than the contig length. Furthermore, bioinformatics analysis was performed using the ALL transcriptome data set. In the Nr annotation, 67.8% of total 65,768 sequences matched perfectly with *E*-values of less than 10^−45^ (Figure S2A), and 61.1% of the matches showed more than 95% similarity (Figure S2B). The species distribution showed that 88.0% had top matches to *F. vesca* subsp. *vesca* genes (Figure S2C), followed by matches to *Prunus persica* (5.9%; Figure S2C). These results indicated that our transcriptome data sets can accurately predict the unigenes potentially useful for further analysis of strawberry species.

In addition, we observed that 72.7% (47,794/65,768, Table 2) consensus sequences from the ALL transcriptome data set showed homology with sequences in the Nr database, whereas 45.8% (30,114/65,768, Table 2) unigenes were similar to proteins in the SWISS-PROT database. A total of 54,813 unigenes [approximately 83.3% (54,813/65,768) of all assembled unigenes were annotated by using combined Nr and SWISS-PROT databases, suggesting that these unigenes have relatively well-conserved functions in our strawberry transcriptome analysis.

GO, an international standardized gene-functional classification system, provides a standardized data by using a strictly defined concept to comprehensively describe the properties of genes and their products. GO was typically used to assign functions to uncharacterized sequences isolated from other organisms. According to GO classifications, a total of 65,768 strawberry unigenes with putative functions assigned to 33,064 unique sequences were categorized into three main GO categories and 55 sub-categories (functional groups; Figure S3A). The Clusters of Orthologous Groups of proteins (COG) database contains classifications of orthologous gene products that can predict and classify possible functions isolated from other species. In this study, the observed strawberry unigenes were searched against the COG database to predict and classify their possible functions. In total, out of 47,794 Nr hits, 17,441 sequences had COG classifications distributed into 25 COG categories (Figure S3B). Pathway-based analyses can further facilitate understanding the potential biological functions of genes. The Kyoto Encyclopedia of Genes and Genomes (KEGG) pathway database comprises information on a systematic analysis of inner-cell metabolic pathways, functions of gene products and their organism-specific variations [50]. To identify the biological pathways in strawberry, we evaluated 26,776 sequences assigned to 128 KEGG pathways (Table S1). These results highlight the considerable potential of Illumina sequencing to discover metabolic pathway genes by using our strawberry transcriptome data set.

### 2.2. Identifying Nine FaTHSF Genes with Full-Length cDNA Sequences in F. × ananassa Duch. cv. Toyonoka

**Figure 2:**
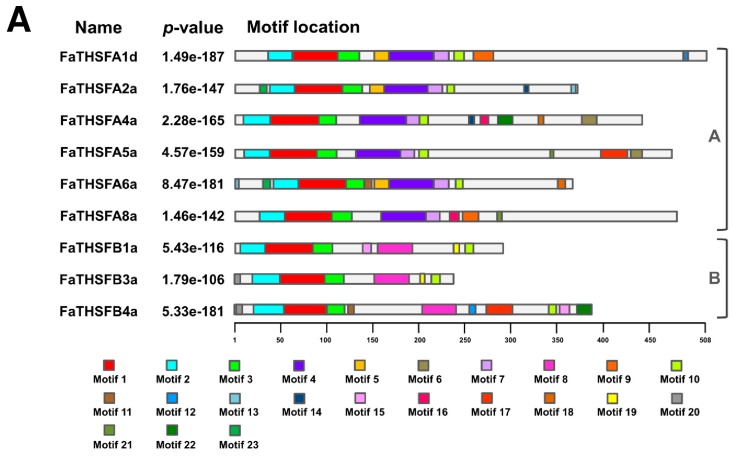

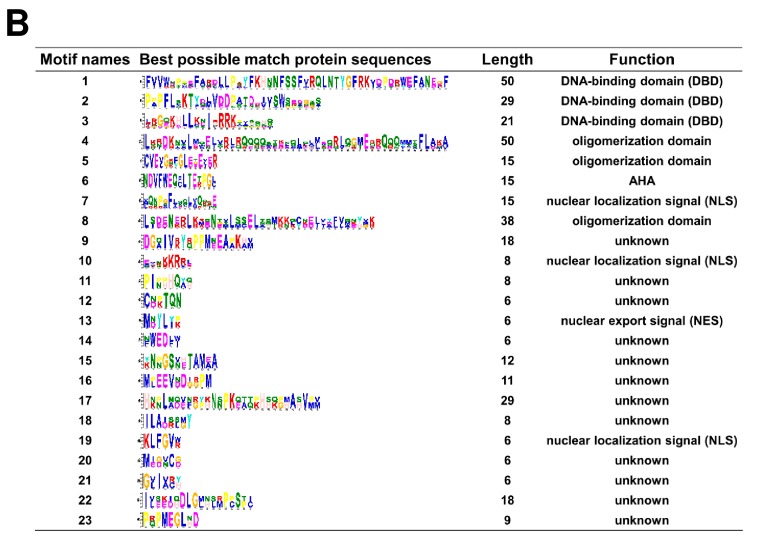
Motifs identified using MEME tools in FaTHSFs. (**A**) The motif location and combined *p*-value of FaTHSFs are shown and denoted by rectangles with different colors. The black closed bracket on the right of the figure shows genes clustering into classes A and B; (**B**) Possible amino acid sequences of Motifs 1–23 identified using MEME tools for FaTHSFs.

Plant HSFs play a central regulatory role in thermotolerance, which subsequently modulates genes encoding HSPs. Plant HSFs are regulated in association mainly with responses to abiotic stress and are associated with primed defense gene activation or pathogen-induced systemic acquired resistance (SAR) [43,51]. We evaluated the conceivable HSF family of *F. × ananassa* Duch. cv. Toyonoka; thus, we not only investigated the transcriptome databases generated in this study, but also the acquired annotation of the identified unigenes. As shown in Table S2, we discovered 31 contigs and 15 unigenes interpreted as HSF proteins, and they appeared to have high homology with *HSF* genes isolated from other plant species, including *Arabidopsis*, rice, and maize. Among these, we further identified nine *FaTHSF* genes containing full-length open reading frames (ORFs; Table S3). The online Multiple EM for Motif Elicitation (MEME) motif search tool was subsequently used and 23 corresponding conserved motifs in FaTHSFs were determined (Figure 2A). The number of motifs in the different FaTHSF proteins showed a high degree of variability. Considering the DBD, Motifs 1, 2, and 3 were basically situated at the N-terminal end of all nine *FaTHSFs* genes (Figure 2A,B). In addition, the presence of different oligomerization domains (the HR-A/B region) determines class A (Motif 4 or 5) or class B (Motif 8) of the FaTHSF family (Figure 2A,B). The class A FaTHSF motif contains other domains, such as CTAD or AHA (Motif 6), NLS (Motifs 7, 10, and 19), and NES (Motif 13; Figure 2A,B). Despite diverse length and sequences of amino acids in each FaTHSF protein, they were all predicted to encompass the DBD and oligomerization domain. Therefore, these and other specific domains result in functional diversity among different FaTHSF proteins.

**Figure 3:**
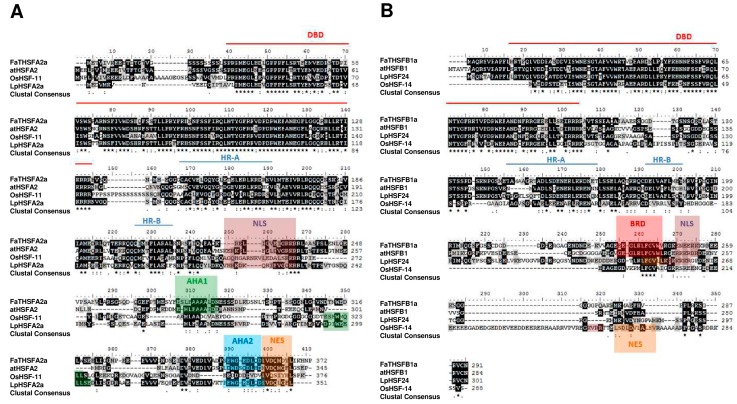
Sequence comparisons of FaTHSFA2a, FaTHSFB1a, and the related HSF proteins. (**A**) Amino acid sequence alignment of the full-length FaTHSFA2a and related HSF proteins, including atHSFA2 (At2g26150, *A. thaliana*), OsHSF-11 (AAQ23058, *O. sativa*), and LpHSFA2a (CAA47870, *L. peruvianum*); (**B**) Amino acid sequence alignment of the full-length FaTHSFB1a and its associated HSF proteins, such as atHSFB1 (At4g36990, *A. thaliana*), OsHSF-14 (AAQ23055, *O. sativa*), and LpHSF24 (P22335, *L. peruvianum*). This sequence alignment was performed by ClustalX 1.8 and BioEdit 7.0 software. Completely and partly conserved amino acids in proteins are shaded in black and gray, respectively. The letters and marks in the alignment are represented as follows: DBD (red line), HR-A and -B (blue line), NLS (highlighted in brown), AHA (highlighted in light green for AHA1 and light blue for AHA2), NES (highlighted in orange), and BRD (highlighted in red).

We specifically selected *FaTHSFA2a* and *FaTHSFB1a* for further investigation not only because they represent class A2 and B1 HSFs, respectively, but also because previous studies have shown that HsfA2 responded strongly to long-term heat stress, accumulating to very high levels in tomato [44], Arabidopsis [45], or rice [46]. In addition, several lines of evidence have revealed that HsfA2 is associated with the expression of Hsps or stress-related non-chaperone encoding genes [44,45]. Thus, these data suggested that HsfA2 probably is one of the strongest heat-associated HSFs. Furthermore, HsfB1a and HsfB2a/b were demonstrated to be related to pathogen resistance in Arabidopsis [43,47,48]. Both FaTHSFA2a and FaTHSFB1a comprise the N-terminal highly conserved DBD and an adjacent oligomerization domain (the HR-A/B region), and their relationship with other *HSF* genes from *Arabidopsis*, rice (*Oryza sativa*), and tomato (*Solanum lycopersicum*) was examined. The *FaTHSFA2a* transcript possesses an ORF of 1119 nucleotides, encoding a protein of 372 amino acids with a predicted molecular weight (*M*w) of 41.95 kDa and an isoelectric point (pI) of 4.93 (Table S3). Amino acid sequence analysis and multiple alignments showed that the deduced amino acid sequences of FaTHSFA2a encompass all critical domains and motifs as those in most HSF families, including the DBD, the HR-A/B region, nuclear localization signal (NLS), nuclear export signal (NES), and a CTAD, which contains AHA1 (ESLFAAAALDN) and AHA2 (SEWGEDLQD). Thus, FaTHSFA2a is similar to atHSFA2 (Figure 3A). By contrast, the *FaTHSFB1a* transcript has an ORF of 873 nucleotides, encoding a protein composed of 290 amino acids with a predicted *M*w of 32.10 kDa and pI of 6.4 (Table S3). Multiple alignment analysis revealed that the deduced amino acid sequences of FaTHSFB1a enclose the DBD, the HR-A/B region, NLS, and a B3 repression domain (BRD; Figure 3B). These domains or motifs were shown to be highly conserved in class B1 HSFs, such as atHSFB1. Therefore, our data suggested that FaTHSFA2a and FaTHSFB1a belong to class A2 and B1 HSFs, respectively.

### 2.3. Classifying FaTHSFA2a and FaTHSFB1a into A2 and B1 HSFs, Respectively, by Phylogenic Analysis

**Figure 4:**
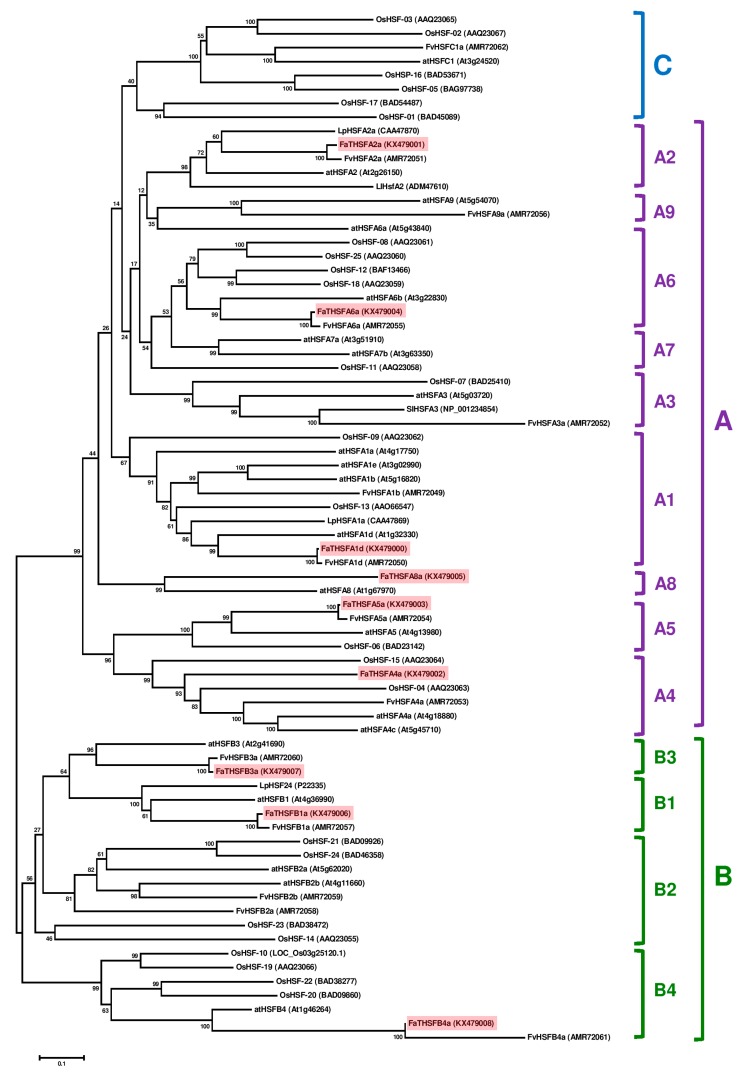
Phylogenetic relationships among the nine full-length *FaTHSF* genes identified in *F. × ananassa* Duch. cv. Toyonoka and other plant HSF proteins. The phylogenetic tree was established by the neighbor joining method by using MAGA 6.0 software. Numbers on major branches indicate bootstrap percentages for 1000 replicate analyses. The tree shows three major clades: class A (A1–A9 subclades), class B (B1–B4 subclades), and class C. Nine unigenes with full-length HSFs identified in this study are highlighted in pink. The abbreviations for the names of different species are as follows: at, *Arabidopsis thaliana*; Fv, *Fragia vesca*; Lp, *Lycopersicon peruvianum*; Os, *Oryza sativa*; and Sl, *Solanum lycopersicum*. The accession numbers for the corresponding genes are provided in parenthesis with the protein names.

To determine the phylogenetic relationships among FaTHSFA2a, FaTHSFB1a, 22 Arabidopsis HSFs, 25 rice HSFs, and five tomato HSFs, a neighbor-joining phylogenetic tree was constructed using MEGA6 [52]. This method basically determines genetic distances according to the sequence identity and similarity and difference among the full-length amino acid sequences of samples. Our phylogenetic analysis results revealed that FaTHSFA2a belongs to class A2 HSFs, with the nearest relative to LpHSFA2a (58.9% identity and 82.8% similarity) and atHSFA2 (55.2% identity and 79.0% similarity; Figure 4). However, FaTHSFB1a belongs to class B1 HSFs, and its nearest relatives are LpHSF24 (58.2% identity and 79.1% similarity) and atHSFB1 (58.3% identity and 80.0% similarity; Figure 4). According to the potential function of class A and B HSFs in heat shock responses and our data, we suggest that FaTHSFA2a acts as a transcriptional activator. By contrast, the putative function of FaTHSFB1a may be involved in the transcriptional repression. Seven class A or B HSFs, containing full-length genes were previously discovered: A1 *HSF* (*FaTHSFA1d*), A4 *HSF* (*FaTHSFA4a*), A5 *HSF* (*FaTHSFA5a*), A6 *HSF* (*FaTHSFA6a*), A8 *HSF* (*FaTHSFA8a*), B3 *HSF* (*FaTHSFB3a*), and B4 *HSF* (*FaTHSFB4a*; Figure 4). Among the wild-type diploid woodland strawberry *F. vesca*, which was composed of 17 classified *FvHSF* genes, namely 11 class A, five class B, and one class C genes, only 14 genes were isolated, including *FvHSFA4b* and *FvHSFA7a* (class A). Hu et al. (2015) could not isolate *FvHSFA8a* from class A HSFs [53]. In our study, other class A subgroup (A3, A7, and A9), class B subgroup (B2), and class C *HSF* genes could not be identified either because our collected tissues were not subjected to heat stress before transcriptome analysis, resulting in lower expression of those genes, or complete lack of those genes in *F. × ananassa* Duch. cv. Toyonoka. However, we were able to identify *HSF* (*FaTHSFA8a*) gene which was only with basal expression when collected tissues were not subjected to heat stress before transcriptome analysis.

Furthermore, phylogenetic analysis from comparison among nine *FaTHSFs*, 14 *FvHSF*s, *Arabidopsis*, rice, and tomato *HSF* genes revealed that *FaTHSFA1d* and *FvHSFA1d* belong to A1 *HSFs* and share 98.8% identity; *FaTHSFA2a* and *FvHSFA2*a are A2 *HSFs* and share 92.9% identity; *FaTHSFA4a* and *FvHSFA4a* are A4 *HSFs* and share 37.5% identity; *FaTHSFA6a* and *FvHSFA6a* are A6 *HSFs* and share 97.0% identity, suggesting they are homologous relatives. Notably, *FaTHSFB1a* and *FvHSFB1a* are B1 *HSFs* and share 96.6% identity, while *FaTHSFB3a* and *FvHSFB3a* belong to B3 *HSFs* and share 97.5% identity. Lastly, *FaTHSFB4a* and *FvHSFB4a* are B4 *HSFs* and share 99.3% identity. Altogether, these data indicate that they are indeed closely-related orthologous homologs. In addition, further overexpression of *FaTHSFA2a* and *FaTHSFB1a* in transgenic *Arabidopsis* enhanced plant resistance to heat stress, indicating that these genes play crucial roles in plants’ thermotolerance by using “gain of function” strategies. Thus, this is the major difference between our studies and those reported by Hu et al. [53]. We mainly focused on functional assays, whereas they intended to discover whether all the HSF-related genes exhibited differential changes at the transcriptional level following heat treatment.

### 2.4. Enhanced Thermotolerance in FaTHSFA2a- and FaTHSFB1a-Overexpressed Transgenic Arabidopsis Plants

**Figure 5:**
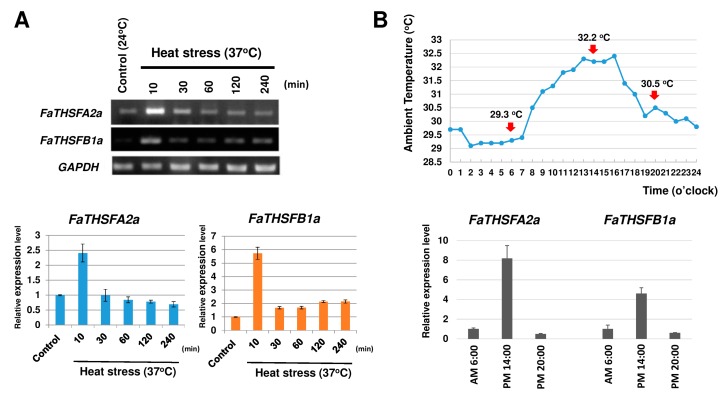
Induction of *FaTHSFA2a* and *FaTHSFB1a* expression under heat stress and the variation in these 2 gene expression were tightly monitored in response to various ambient temperatures within 24 h. (**A**) Induction of *FaTHSFA2a* and *FaTHSFB1a* expression under heat stress (37 °C). The garden strawberry was grown at 24 °C as a control or at 37 °C to subject it to heat stress for different time periods. The leaf tissues of the control and heat stress-treated plants were subsequently collected, and the total RNA was extracted thereafter at 10, 30, 60, 120, and 240 min. Semi-quantitative RT-PCR analysis was performed for each sample collected at different time points and normalized with GAPDH (=1). Error bars indicate standard deviation (*n* = 3); (**B**) Temperature variation in the environment where the garden strawberry was cultivated was recorded once every hour within 24 h on the same day the leaf samples were collected. The leaf tissues were collected and the total RNA was extracted at 6 am (29.3 °C), 2 pm (32.2 °C), and 8 pm (30.5 °C), respectively, and real-time qRT-PCR analysis was performed and normalized with GAPDH (=1). Error bars represent standard deviation (*n* = 3).

Semi-quantitative RT-PCR revealed that *FaTHSFA2a* and *FaTHSFB1a* expression was markedly upregulated, to approximately 2.4-fold for *FaTHSFA2a* and 5.7-fold for *FaTHSFB1a* compared with the control, when strawberry leaf tissues were subjected to heat shock at 37 °C for 10 min. By contrast, *FaTHSFA2a* and *FaTHSFB1a* expression was low when leaf tissues were subjected to a control temperature of 24 °C (Figure 5A). In addition, strawberry plants were grown in the field, and the temperature variation was recorded at different time periods, once every hour, particularly at 6 am (29.3 °C), 2 pm (32.2 °C), and 8 pm (30.5 °C). Strawberry leaf tissues were subsequently collected at these time points. The total RNA was extracted, and the samples were subjected to real-time quantitative RT (qRT)-PCR analysis. As shown in Figure 5B, *FaTHSFA2a* expression was increased by approximately 8.2-fold from time points 1 to 2 when the temperature was increased from 29.3 °C to 32.2 °C. However, the expression decreased to the original level from time points 2 to 3 when the temperature was decreased to 30.5 °C. Similarly, *FaTHSFB1a* was approximately 4.6-fold augmented from time points 1 to 2, thereafter declining at time point 3. Thus, these data indicated that *FaTHSFA2a* and *FaTHSFB1a* expression was induced by heat stress in a temperature-dependent manner. Notably, the induction decreased at a later stage of heat shock treatment.

**Figure 6:**
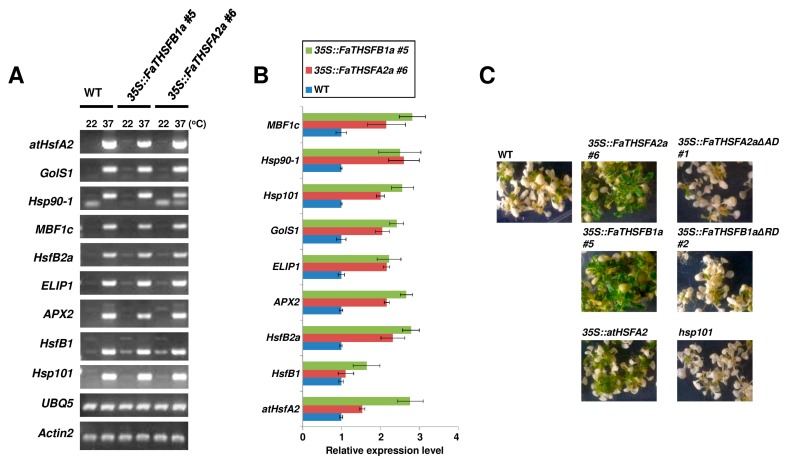
Roles of *FaTHSFA2a* and *FaTHSFB1a* in basal thermotolerance. (**A**) Up-regulated gene expression in *35S::FaTHSFA2a#6* and *35S::FaTHSFB1a#5* transgenic plants were observed after heat stress. Total RNAs were extracted from 10-day-old plants grown in MS medium at 22 °C and subsequently were either exposed to 22 °C as a control or moved to a 37 °C incubator for 1 h as heat stress treatment. The extracted RNAs were further used in semi-quantitative RT-PCR assays for wild-type (WT), *35S::FaTHSFA2a#6*, and *35S::FaTHSFB1a#5* plants by using gene-specific primer sets (Table S4). In this assay, *Ubiquitin 5* (*UBQ5*) and *Actin2* (*ACT2*) were used as the loading controls. Similar results were observed for *35S::FaTHSFA2a#4* and *#5* and *35S::FaTHSFB1a#3* and *#4* transgenic plants; (**B**) Analysis of mRNA levels in 10-days-old WT, *35S::FaTHSFA2a#6*, *35S::FaTHSFA2aΔAD#1*, *35S::FaTHSFB1a#5*, and *35S::FaTHSFB1aΔRD#2* at 22 °C by using gene-specific primer sets (Table S4). The levels of mRNA were determined by real-time qRT-PCR, and normalized with *Actin2/8* (=1). Error bars indicate the standard deviation (*n* = 3); (**C**) Thermotolerance evaluation of positive transgenic *Arabidopsis* lines with the overexpression of *FaTHSFA2a#6*, *FaTHSFA2aΔAD#1*, *FaTHSFB1a#5*, and *FaTHSFB1aΔRD#2*. Ten-day-old WT and transgenic plants exposed to heat stress at 45 °C for 1 h and subsequently recovered at 22 °C for 7 days were evaluated for their greening cultivars and photographed. More than 20 plants were analyzed in each experimental set (*n* = 3). Similar results were observed from triplicate experiments.

To explore the in vivo function of FaTHSFA2a and FaTHSFB1a, transgenic plants were generated to overexpress full-length *FaTHSFA2a* and *FaTHSFB1a* genes under the control of cauliflower mosaic virus (CaMV) 35S promoter. Among T2 selection lines, six independent lines (#1–#6) for *35S::FaTHSFA2a* transgenic plants with a higher expression of *FaTHSFA2a* transcripts and six independent lines (#1–#6) for *35S::FaTHSFB1a* transgenic plants with an increased expression of *FaTHSFB1a* transcripts were selected for further observation (Figure S4). Semi-quantitative RT-PCR analysis was performed for transgenic plants grown under non-heat stress (22 °C) and heat stress (37 °C) conditions. Several HSPs, HSFs, and stress-associated genes, including *Hsp101*, *MBF1C*, and *ELIP* were upregulated and detected in *35S::FaTHSFA2a* and *35S::FaTHSFB1a* transgenic plants without heat stress treatment (22 °C), whereas they were barely detected in wild-type plants grown under the same conditions (Figure 6A). We also obtain similar results confirmed by real-time qPCR analysis (Figure 6B). As expected, these stress-associated genes exhibited a heat-inducible expression pattern in the wild-type plants grown at 37 °C (Figure 6A). Moreover, our data revealed that these stress-associated genes were upregulated in *35S::FaTHSFA2a* and *35S::FaTHSFB1a* transgenic plants during heat stress treatment, probably because of the increased expression of endogenous *HSFA2* and other members of the HSF family (*HSFB1* and *HSFB2a*) under heat stress (Figure 6A,B).

To examine whether the biological function of the C-terminal trans-activation domain in FaTHSFA2a and trans-repression domain in FaTHSFB1a were required for the thermotolerance of plants, we generated two transgenic plants overexpressing *FaTHSFA2aΔAD* (containing 1-233aa of FaTHSFA2a, with a deleted trans-activation domain, named 35S::*FaTHSFA2aΔAD*) and *FaTHSFB1aΔRD* (containing 1-232aa of FaTHSFB1a, with a deleted trans-repression domain, named 35S::*FaTHSFB1aΔRD*). RT-PCR confirmed a higher expression of these truncated transcripts among T2 selection lines compared with that in wild-type plants (Figure S4). The basal thermotolerance of *35S::FaTHSFA2a*, *35S::FaTHSFA2aΔAD*, *35S::FaTHSFB1a*, and *35S::FaTHSFB1aΔRD* plants were subsequently compared with that of wild-type plants. Under heat stress (45 °C), most *35S::FaTHSFA2aΔAD*, *35S::FaTHSFB1aΔRD*, and *hsp101* mutant-like wild-type plants died after 7 days, whereas *35S::FaTHSFA2a* and *35S::FaTHSFB1a* plants, such as *35S::atHSF2A* survived under the same condition (Figure 6C). Thus, our results support the notion that the C-terminal functional domain of both FaTHSFA2a- and FaTHSFB1a-containing plants is essential for their increased thermotolerance in response to heat stress. Furthermore, our data revealed that the overexpression of *FaTHSFA2a* and *FaTHSFB1a* led to the constitutive expression of their downstream heat-stress-responsive genes in *Arabidopsis* transgenic plants, which may also contribute to the enhanced thermotolerance observed in the transgenic plants compared with wild-type plants.

### 2.5. Nuclear Localization of FaTHSFA2a and FaTHSFB1a Suggesting Their Roles as Transcription Factors

**Figure 7:**
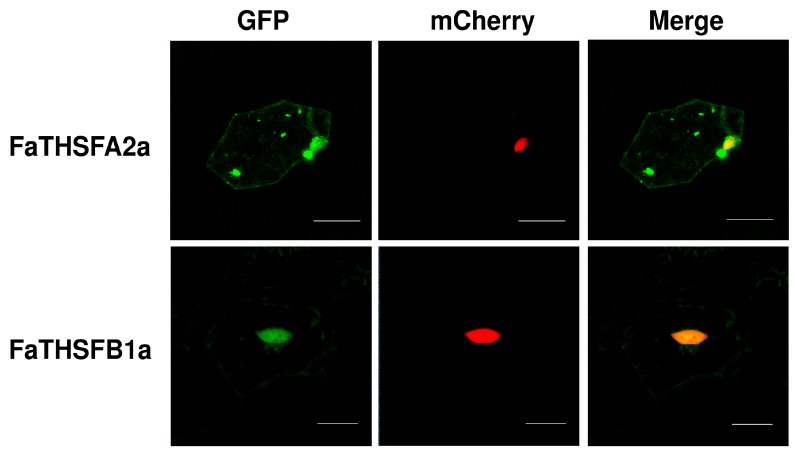
Subcellular localization of fluorescent GFP–FaTHSFA2a and GFP–FaTHSFB1a proteins. Recombinant plasmids harboring GFP–FaTHSFA2a and GFP–FaTHSFB1a, whose gene expression is driven by the CaMV35S promoter was transiently expressed in flower lips of orchids. These two recombinant plasmids (35S::GFP–FaTHSFA2a and 35S::GFP–FaTHSFB1a) were delivered into flower lips of orchids by using the particle bombardment method. The NLS domain of VirD2 fused with mCherry was used as the nuclear marker in this study. Overlays (merge) are shown in the extreme right column. The bar in each figure represents 20 μm.

According to our study, the unique functions of FaTHSFA2a and FaTHSFB1a proteins can act as HSFs. Therefore, these proteins were predicted to be located in the nucleus. To investigate the subcellular localization of FaTHSFA2a and FaTHSFB1a proteins, protoplasts prepared from flower lips of orchids were used for the transformation of *GFP–FaTHSFA2a* (*35S::GFP–FaTHSFA2a*) and *GFP–FaTHSFB1a* (*35S::GFP–FaTHSFB1a*) fusion genes, respectively, by particle bombardment. Our results detected the fluorescent signals of the GFP–FaTHSFA2a and GFP–FaTHSFB1a proteins mainly in the nucleus compared with the control GFP whose fluorescence was located in both nucleus and cytosol (Figure 7). These data revealed that FaTHSFA2a and FaTHSFB1a proteins were not confined in the nucleus; they can be transferred into the cytoplasm where they might conceivably perform other functions. Our results are in concordance with previous data reported by Hu et al. (2015) [53]; they clearly detected most FvHSF–GFP proteins in the nucleus. However, some FvHSF–GFP proteins, including FvHSF2a, FvHSF3a, FvHSF4a, FvHSF5a, FvHSF2b, and FvHSFC1a, remained detectable in the cytosol [53]. Thus, our results revealed that FaTHSFA2a and FaTHSFB1a proteins have the capability to enter the nucleus and putatively function as transcriptional regulators.

### 2.6. FaTHSFA2a Exhibited Trans-Activation Function, Whereas FaTHSFB1a Showed Trans-Repression Activity

**Figure 8:**
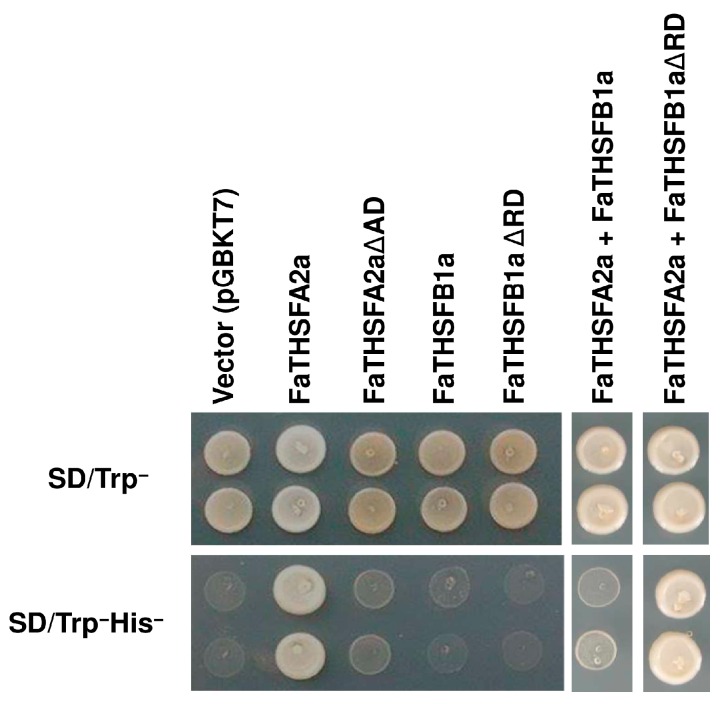
Analysis for measuring trans-activation activity in yeast cells. The full-length constructs of FaTHSFA2a and FaTHSFB1a were fused to GAL4 DBD and expressed in the yeast strain AH109. The transformed yeast cells were grown in non-selective media with histidine (SD/Trp^−^) or selective media without histidine (SD/Trp^−^His^−^), followed by incubation at 30 °C for 3 days. Two independent transformants were selected from each construct in the presence of histidine (SD/Trp^−^). Subsequently, yeast cells containing either single construct or cotransformed constructs were spotted on the media with (SD/Trp^−^) or without histidine (SD/Trp^−^His^−^). The pGBKT7 vector transformed into yeast cells was used as the negative control.

According to the deduced amino acid sequence and phylogenetic analysis, FaTHSFA2a belongs to the Class A2 HSF subgroup, whereas FaTHSFB1a is classified into the Class B1 HSF subgroup. To examine whether FaTHSFA2a possesses a CTAD similar to other Class A HSFs and FaTHSFB1a contains a BRD similar to other Class B HSFs in their gene structure, we performed a yeast one-hybrid assay. The full-length or C-terminal truncated fragments of FaTHSFA2a and FaTHSFB1a were fused to the GAL4 DBD and transformed into the yeast reporter strain AH109 harboring *HIS3* reporter genes. The activity of *HIS3* reporter genes was confirmed by a viability test with a selective medium lacking histidine. In the absence of histidine, the yeast cells transformed with the construct containing the full-length ORF of FaTHSFA2a survived. By contrast, yeast cells transformed with the C-terminal deletion of the *FaTHSFA2a* construct (no CTAD) could not survive under the same condition (Figure 8). By contrast, yeast cells transformed with either the full-length ORF or C-terminal deletion construct containing no BRD of *FaTHSFB1a*, could not survive in the selective medium without histidine (Figure 8). Subsequently, we co-transformed yeast cells containing the full-length ORF of *FaTHSFA2a* with either *FaTHSFB1a* or *FaTHSFB1aΔRD*. In contrast to the survival of yeast cells harboring *FaTHSFA2a* in the medium without histidine, yeast cells harboring both full-length *FaTHSFA2a* and *FaTHSFB1a* could not survive. However, yeast cells harboring *FaTHSFA2a* and subsequently transformed with *FaTHSFB1aΔRD* survived in the aforementioned medium. These results revealed that *FaTHSFA2a* functions as a transcriptional activator, exhibiting trans-activation activity similar to that of other class A HSFs, such as *HsfA1a*, *HsfA1b*, *HsfA1d*, *HsfA1e*, and *HsfA2* [45,54,55,56,57,58]. By contrast, *FaTHSFB1a* acts as a transcriptional repressor, which expresses trans-repression activity similar to that of other *HsfB1* identified in *Arabidopsis*, tomato, and soybean [25,26,48,59].

## 3. Materials and Methods

### 3.1. Plant Materials and RNA Extraction

In this study, we used the octoploid strawberry *F. × ananassa* Duch. cv. *Toyonoka*, which needs short-day and low temperature (cold) conditions to accelerate flower bud initiation [60,61]. Strawberry seedlings were grown in the field and widely planted in Industrial Technology Research Institute. All plants were watered daily and fertilized weekly. We selected plants with similar height, and their crown diameter was moved, and cultivated in the growth chamber. To reduce the microclimate effects in the growth chamber, all plants placed in each layer inside the chamber were rotated once weekly. The total RNA was extracted from strawberry tissues, namely leaves, floral buds, fully bloomed flowers, immature fruits, roots, and vegetative, and inflorescence meristems that were separately harvested and immediately frozen in liquid nitrogen, followed by storage at −80 °C until use. To perform Semi-quantitative RT-PCR for *FaTHSFA2a* and *FaTHSFB1a* expression, strawberry leaf tissues were subjected to heat shock at 37 °C for 10 min. All sample RNA were extracted with cetyl trimethylammonium bromide based buffer, as previously reported [62]. The quality and quantity of RNA extracts were evaluated using the Nanodrop ND-1000 spectrophotometer (Thermo Scientific, Wilmington, DE, USA) and visualized through 1% agarose gel electrophoresis under denaturing conditions.

### 3.2. cDNA Preparation, Sequencing and De Novo Assembly

Equal amounts of total RNA from each strawberry tissue were mixed for the subsequent steps of the following experiments. Poly-A-containing mRNAs were purified from the mixed total RNA samples. The paired-end cDNA library was synthesized using the Genomic Sample Prep kit (Illumina, San Diego, CA, USA), according to the manufacturer’s instructions. The sequencing cDNA library was constructed through PCR amplification and directly sequenced using the Illumina Genome Analyzer according to the manufacturer’s instructions (Genomics BioSci & Tech., Taipei, Taiwan). The raw sequencing data were subsequently filtered to remove low-quality sequences, including ambiguous nucleotides, adaptor sequences, and repeat sequences. The following de novo transcriptome assemblies of these short reads were performed using the SOAP de novo program [63]. To determine the consensus coding sequence (CDS) and sequence direction of the unigenes, these unigenes were analyzed using the BLASTX program (NCBI, Bethesda, MD, USA) with Nr, SWISS-PROT, KEGG, and COG databases [63]. The CDS of unigenes which were aligned to none of the aforementioned databases, were subsequently determined from the coding regions and sequence direction prediction by using ESTScan software [64]. Our transcriptome data sets are available at the NCBI Sequence Read Archive (SRA), under the accession numbers SRX1895539 and SRX1895540 for OF and DU transcriptome data sets, respectively.

### 3.3. Functional Annotation and Classification

Unigene annotations provide functional information, including protein sequence similarities, GO, Clusters of Orthologous Groups of proteins (COG) clusters, and Kyoto Encyclopedia of Genes and Genomes (KEGG) pathway data. A sequence similarity search was conducted using the NCBI Nr, SWISS-PROT, COG, and KEGG pathway databases by using the BLASTX algorithm specifying *E*-values of less than 10^−5^. Thus, unigene annotations may identify genes with their potential expression patterns and functional annotations. From the NCBI Nr protein database, we obtained GO annotations of these assembled unigenes by using the BLAST2GO program (https://www.blast2go.com/) [65]. Thereafter, we utilized WEGO software (Genomics BioSci & Tech., Taipei, Taiwan) to conduct GO functional classifications for all identified unigenes, and explored the macro-distribution of gene functions for this strawberry species [65]. Here, these strawberry unigenes with Nr annotation obtained using the BLAST2GO program [65], followed by WEGO software [66], were subjected to GO functional classifications, and the major distribution of the gene functions of this species was determined. Furthermore, we performed metabolic pathway analysis by using the KEGG database and related software applications (http://www.genome.jp/kegg/kegg4.html) [20]. Annotation with COGs and KEGG pathways was performed by searching with the BLASTX program against the COG [67] and KEGG [68] databases, with an E-value threshold of 10^−5^.

### 3.4. Reverse Transcription Polymerase Chain Reaction (RT-PCR) and Real-Time RT-PCR Analysis

First strand cDNA was synthesized using 2 μg of total RNA, an oligo dT primer, and an ImProm-II^TM^ reverse transcription system (Promega, Madison, WI, USA) according to the manufacturer’s instructions. Samples from each reaction (1 μL) were subsequently used in a 20-μL premix PCR mixture containing Platinum^®^ Taq polymerase (Invitrogen, Carlsbad, CA, USA) and gene-specific primer sets (Table S4). For RT-PCR, amplification was continuously performed for 26 cycles, with each cycle at 94 °C for 30 s, 55 °C for 30 s, and 72 °C for 30 s. The PCR product was collected and analyzed through agarose gel electrophoresis, followed by ethidium bromide staining. By contrast, real-time PCR was conducted for 40 cycles by using 1 μL of cDNA as the template and the CFX-96_Real Time system with SYBR Green Master Mix (Toyobo, Osaka, Japan). Subsequently, real-time RT-PCR data were analyzed using CFX Manager V2.1 software (Bio-Rad, Hercules, CA, USA). All sample data were normalized to tubulin or glyceraldehyde-3-phosphate dehydrogenase mRNA levels. The primer sets used for RT-PCR analysis are listed in Table S4.

### 3.5. Construction of 35S::FaTHSFA2a, 35S::FaTHSFA2aΔAD, 35S::FaTHSFB1a and 35S::FaTHSFB1aΔRD Recombinant Plasmids

Both full-length and C-terminal-truncated fragments of FaTHSFA2a and FaTHSFB1a coding regions (*FaTHSFA2a*, *FaTHSFB1a* and *FaTHSFA2aΔAD*, and *FaTHSFB1aΔRD*) were amplified from Toyonoka strawberry cDNAs through PCR with the gene-specific primer sets containing 5′-BamHI or 3′-SpeI recognition sites (Table S4). These primer sets were specifically designed to facilitate the cloning of these cDNAs by using Platinum^®^ Taq DNA polymerase (Invitrogen). The PCR product was subsequently purified using a purification column (BIOKIT, Miaoli, Taiwan) according to the manufacturer’s instructions, cloned into the pGEM-T easy vector (Promega, Madison, WI, USA), and further sequenced to confirm the DNA sequences of the selected clones. T-vector with an appropriate insertion orientation was selected through the release of the respective full-length and C-terminal truncated cDNA fragment through BamHI and SpeI digestion and further subcloned from pGEM-T into the binary vector pCambia1390-35S vector (CAMBIA, Brisbane, Australia) between the BamHI and SpeI sites downstream of the CaMV 35S promoter. Gene-specific PCR combined with restriction enzyme digestion analysis was used to confirm the pCambia1390-35S vector harboring 35S::FaTHSFA2a, 35S::FaTHSFA2aΔAD, 35S::FaTHSFB1a, or 35S::FaTHSFB1aΔRD. *Agrobacterium tumefaciens* Strain GV3101 was used as the host for the resultant constructs.

### 3.6. *Arabidopsis thaliana* Transformation and Phenotypic Analysis

The aforementioned constructs harboring 35S::FaTHSFA2a, 35S::FaTHSFA2aΔAD, 35S::FaTHSFB1a, or 35S::FaTHSFB1aΔRD were transformed into wild-type Arabidopsis plants (ecotype: Col-0) by using the floral-dipping method [69]. The seeds were harvested and surface-sterilized with 25% sodium hypochlorite solution. These transgenic plants were selected on solid half MS medium containing 30 μg/mL hygromycin. The plates were sealed with 3 M micropore tape and placed vertically in a growth chamber (CH-202, CHIN-HSIN, Taipei, Taiwan), with a long-day condition (16-h light/8-h dark cycle), and 120 Lmol·m^−2^·s^−1^ photo flux density at 22 ± 2 °C for 12 days before transplanting to soil. The ectopic gene expression of FaTHSFA2a, FaTHSFA2aΔAD, FaTHSFB1a, and FaTHSFB1aΔRD was confirmed through RT-PCR by using gene-specific primer sets (Table S4). Each transgenic whole plant was photographed using a digital camera (Canon, PowerShot A640, Tokyo, Japan). Young seedlings of wild-type and all transgenic plants were viewed under a dissecting microscope (Leica, MS5, Heerbrugg, Switzerland), and all image data were collected using a digital camera (Canon, PowerShot S80, Tokyo, Japan) under a light-field.

### 3.7. Heat Stress Assays

For both basal thermotolerance and acquired thermotolerance assays, wild-type and transgenic plants were cultivated on a half MS medium in plastic Petri dishes and incubated in growth chambers (CH-202, CHIN-HSIN, Taipei, Taiwan). In basal thermotolerance assays, 5-day-old plants were subjected to 45 °C for 90 min, and the temperature was subsequently reduced to 22 °C [70]. Alternatively, 10-day-old plants were grown at 45 °C for 60 min and transferred to a 22 °C incubator. Following continuous growth at 22 °C for 5 days, these plants were photographed [70]. The number of survived plants was counted and recorded daily after the heat stress treatment.

### 3.8. Subcellular Localization

Both full-length FaTHSFA2a and FaTHSFB1a cDNAs were PCR-amplified with a pair of primers with attB1/B2 sites (Table S4) and cloned into the pDONR221 vector by using Gateway BP Clonase II Enzyme Mix (Invitrogen, Carlsbad, CA, USA). Each construct was subsequently recombined as an N-terminal fusion of GFP into the Gateway destination binary vector pK7WGF2 (Functional Genomics Division of the Department of Plant Systems Biology, Gent, Belgium), yielding 35S::GFP–FaTHSFA2a and 35S::GFP–FaTHSFB1a by an attL× attR recombination reaction (Invitrogen, Carlsbad, CA, USA). These GFP fusion constructs were isolated and transformed into floral lips of orchids by bombardment transformation [71]. A Zeiss LSM 510 META laser-scanning confocal microscope using an LD C-Apochromat 409/1.1 W objective lens was subsequently used to observe the fluorescence emitted in the transformed cells, as previously described [71].

### 3.9. Transcriptional Activation or Repression Activity in Yeast

For measuring trans-activation or trans-repression activity, both full-length coding regions and C-terminal truncated fragments of FaTHSFA2a and FaTHSFB1a were amplified through PCR with a set of primers containing attB1/B2 sites and cloned into the pDONR221 vector by using Gateway BP Clonase II Enzyme Mix (Invitrogen, Carlsbad, CA, USA). Subsequently, the cloned cDNAs were transferred into the pGBKT7 BD vector by using Gateway LR Clonase II Enzyme Mix (Invitrogen, Carlsbad, CA, USA) [72]. Each BD-construct was introduced into *Saccharomyces cerevisiae* strain AH109 and tested for its ability to activate or repress the transcription of HIS3 reporter genes by using a small-scale yeast transformation protocol (Clontech, Palo Alto, CA, USA). The transformed yeast cells were spotted on SD/Trp^−^ and SD/His^−^/Trp^−^ media. The plates were subsequently incubated at 30 °C for 3 days, and HIS3 activity was confirmed on histidine-lacking solid medium.

## 4. Conclusions

The assembled transcriptome database in the present study provides a foundation for the molecular genetics and functional genomics insights required to manipulate desirable agronomic traits or molecular breeding of garden strawberry. We used Illumina second-generation sequencing technology, and surveyed the transcriptome of *F. × ananassa* Duch. cv. Toyonoka. We combined two transcriptome data sets, and further assembled 65,768 unigenes and annotated 54,813 of these unigenes. This comprehensive coverage indicates the identification of almost all known genes from the major metabolic pathways responsible for strawberry development during different stages. Through bioinformatics analysis, we identified nine *FaTHSF* genes with full-length cDNAs (five contigs and four unigenes) from 31 contigs and 15 unigenes interpreted as HSF proteins. Classification of these genes was determined according to the sequence alignments, phylogenetic relationships, and expression analysis. *FaTHSFA2a* and *FaTHSFB1a* were specifically selected due to their representative roles as class A2 and B1 HSFs, respectively, and previous studies have revealed their crucial activities as strong heat-associated HSF proteins [46,47,48,49,50,51]. So, they were further isolated, characterized, and found up-regulated at the transcription level under heat stress. Notably, when functioning as transcription factors, *FaTHSFA2a* and *FaTHSFB1a* activated downstream heat stress-associated genes. In addition, enhanced thermotolerance was observed in *FaTHSFA2a*- and *FaTHSFB1a*- overexpressed transgenic *Arabidopsis* plants. Notably, yeast one-hybrid analysis revealed that FaTHSFA2a performs its trans-activation function by using the CTAD, and FaTHSFB1a exerts trans-repression activity through BRD. Thus, our data suggest that *FaTHSFA2a* and *FaTHSFB1a* function as crucial transcriptional activator and repressor, respectively, in the heat signaling pathways in garden strawberry. Consequently, it would be beneficial to employ the transformation of genes encoding FaTHSFA2a and FaTHSFB1a in plants to increase the thermotolerance of cultivated strawberry or other Rosaceae species in molecular-assisted breeding. This study also demonstrates that NGS technology is a rather rapid and cost-effective method for de novo transcriptome analysis of non-model plants, for which genomic information remains unavailable.

## Figures and Tables

**Table 1:** Output statistics of sequencing.

Samples	Total Clean Reads ^1^	Total Clean Nucleotides (nt) ^1^	Q20 Percentage ^2^	N Percentage ^3^	GC Percentage ^4^
OF	47,551,534	4,279,638,060	95.98%	0.00%	48.16%
DU	49,032,098	4,412,888,820	96.99%	0.00%	47.45%

^1^ Total reads and total nucleotides are actually clean reads and clean nucleotides. The total nucleotides should be greater than contract provision; ^2^ The Q20 percentage is the proportion of nucleotides with a quality value higher than 20; ^3^ The N percentage is the proportion of unknown nucleotides in clean read; ^4^ The GC percentage is the proportion of guanidine and cytosine nucleotides among total nucleotides.

**Table 2:** Statistics of the assembly quality.

Classification	Sample	Total Number	Total Length (nt)	Mean Length (nt)	N50 ^1^
Contig	OF	117,813	41,433,873	352	740
	DU	122,261	43,572,190	356	805
Unigene	OF	65,164	45,910,155	705	1218
	DU	72,914	50,183,039	688	1155
	All	65,768	57,422,693	873	1387

^1^ N50: length of the smallest transcripts in the set that contain the fewest (largest).

## Supplementary Materials

Supplementary materials can be found at www.mdpi.com/1422-0067/17/12/2130/s1.
