# Supplementary material for: Identification of Heat Shock Transcription Factor Genes Involved in Thermotolerance of Octoploid Cultivated Strawberry
Source: Int J Mol Sci. 2016 Dec 17;17(12):2130. doi: 10.3390/ijms17122130 (PMC5187930; doi:10.3390/ijms17122130)
Supplement: Supplementary file 1 [file ijms-17-02130-s001.zip › ijms-151236-Supplementary Materials/ijms-151236-Supplementary.pdf]

Supplementary Materials: Identification of Heat Shock Transcription Factor Genes Involved in Thermotolerance of Octoploid Cultivated Strawberry

Wan-Yu Liao, Lee-Fong Lin, Jing-Lian Jheng, Chun-Chung Wang, Jui-Hung Yang and Ming-Lun Chou

Table S1. Categorization of strawberry unigenes to KEGG biochemical pathways.

Number	Pathway	All Genes with Pathway Annotation (26776)	Pathway ID
1	Metabolic pathways	6911 (25.81%)	ko01100
2	Biosynthesis of secondary metabolites	2998 (11.2%)	ko01110
3	Plant-pathogen interaction	1817 (6.79%)	ko04626
4	Endocytosis	1598 (5.97%)	ko04144
5	Glycerophospholipid metabolism	1497 (5.59%)	ko00564
6	Ether lipid metabolism	1336 (4.99%)	ko00565
7	Plant hormone signal transduction	1297 (4.84%)	ko04075
8	RNA transport	1183 (4.42%)	ko03013
9	Spliceosome	1006 (3.76%)	ko03040
10	Starch and sucrose metabolism	984 (3.67%)	ko00500
11	Purine metabolism	863 (3.22%)	ko00230
12	Pyrimidine metabolism	799 (2.98%)	ko00240
13	Protein processing in endoplasmic reticulum	710 (2.65%)	ko04141
14	Pentose and glucuronate interconversions	628 (2.35%)	ko00040
15	RNA polymerase	598 (2.23%)	ko03020
16	mRNA surveillance pathway	597 (2.23%)	ko03015
17	Ribosome	540 (2.02%)	ko03010
18	Phenylpropanoid biosynthesis	477 (1.78%)	ko00940
19	RNA degradation	468 (1.75%)	ko03018
20	Ribosome biogenesis in eukaryotes	453 (1.69%)	ko03008
21	Ubiquitin mediated proteolysis	431 (1.61%)	ko04120
22	Zeatin biosynthesis	360 (1.34%)	ko00908
23	Glycolysis / Gluconeogenesis	344 (1.28%)	ko00010
24	ABC transporters	319 (1.19%)	ko02010
25	Flavonoid biosynthesis	302 (1.13%)	ko00941
26	Oxidative phosphorylation	296 (1.11%)	ko00190
27	Amino sugar and nucleotide sugar metabolism	292 (1.09%)	ko00520
28	Stilbenoid, diarylheptanoid and gingerol biosynthesis	275 (1.03%)	ko00945
29	Nucleotide excision repair	262 (0.98%)	ko03420
30	Terpenoid backbone biosynthesis	250 (0.93%)	ko00900
31	Phagosome	220 (0.82%)	ko04145
32	Circadian rhythm-plant	219 (0.82%)	ko04712
33	Galactose metabolism	209 (0.78%)	ko00052
34	Phenylalanine metabolism	206 (0.77%)	ko00360
35	Limonene and pinene degradation	205 (0.77%)	ko00903
36	Carotenoid biosynthesis	205 (0.77%)	ko00906
37	Pyruvate metabolism	203 (0.76%)	ko00620
38	Homologous recombination	200 (0.75%)	ko03440
39	Glutathione metabolism	194 (0.72%)	ko00480
40	Tyrosine metabolism	185 (0.69%)	ko00350
41	Cysteine and methionine metabolism	182 (0.68%)	ko00270
42	Fructose and mannose metabolism	181 (0.68%)	ko00051
43	Peroxisome	180 (0.67%)	ko04146
44	Phosphatidylinositol signaling system	179 (0.67%)	ko04070
45	Ascorbate and aldarate metabolism	179 (0.67%)	ko00053
46	Arginine and proline metabolism	176 (0.66%)	ko00330
47	Basal transcription factors	172 (0.64%)	ko03022
48	Inositol phosphate metabolism	172 (0.64%)	ko00562

Table S1. *Cont.*

Number	Pathway	All Genes with Pathway Annotation (26776)	Pathway ID
49	Other glycan degradation	171 (0.64%)	ko00511
50	Cutin, suberine and wax biosynthesis	169 (0.63%)	ko00073
51	Aminoacyl-tRNA biosynthesis	169 (0.63%)	ko00970
52	Cyanoamino acid metabolism	166 (0.62%)	ko00460
53	Glycerolipid metabolism	159 (0.59%)	ko00561
54	DNA replication	156 (0.58%)	ko03030
55	Glycosylphosphatidylinositol (GPI)-anchor biosynthesis	154 (0.58%)	ko00563
56	Carbon fixation in photosynthetic organisms	153 (0.57%)	ko00710
57	Mismatch repair	152 (0.57%)	ko03430
58	Fatty acid metabolism	152 (0.57%)	ko00071
59	Flavone and flavonol biosynthesis	152 (0.57%)	ko00944
60	Pentose phosphate pathway	147 (0.55%)	ko00030
61	Base excision repair	146 (0.55%)	ko03410
62	alpha-Linolenic acid metabolism	140 (0.52%)	ko00592
63	Porphyrin and chlorophyll metabolism	135 (0.5%)	ko00860
64	Glycine, serine and threonine metabolism	133 (0.5%)	ko00260
65	Citrate cycle (TCA cycle)	133 (0.5%)	ko00020
66	Alanine, aspartate and glutamate metabolism	132 (0.49%)	ko00250
67	Ubiquinone and other terpenoid-quinone biosynthesis	130 (0.49%)	ko00130
68	Glyoxylate and dicarboxylate metabolism	123 (0.46%)	ko00630
69	Regulation of autophagy	119 (0.44%)	ko04140
70	Protein export	116 (0.43%)	ko03060
71	Valine, leucine and isoleucine degradation	116 (0.43%)	ko00280
72	Diterpenoid biosynthesis	111 (0.41%)	ko00904
73	Propanoate metabolism	103 (0.38%)	ko00640
74	N-Glycan biosynthesis	102 (0.38%)	ko00510
75	Phenylalanine, tyrosine and tryptophan biosynthesis	99 (0.37%)	ko00400
76	Brassinosteroid biosynthesis	95 (0.35%)	ko00905
77	Brassinosteroid biosynthesis	95 (0.35%)	ko00905
78	Proteasome	93 (0.35%)	ko03050
79	Sphingolipid metabolism	92 (0.34%)	ko00600
80	Biosynthesis of unsaturated fatty acids	90 (0.34%)	ko01040
81	β-Alanine metabolism	84 (0.31%)	ko00410
82	SNARE interactions in vesicular transport	84 (0.31%)	ko04130
83	Photosynthesis	83 (0.31%)	ko00195
84	Isoquinoline alkaloid biosynthesis	81 (0.3%)	ko00950
85	Tryptophan metabolism	79 (0.3%)	ko00380
86	Sesquiterpenoid and triterpenoid biosynthesis	77 (0.29%)	ko00909
87	Natural killer cell mediated cytotoxicity	75 (0.28%)	ko04650
88	Steroid biosynthesis	72 (0.27%)	ko00100
89	Lysine degradation	72 (0.27%)	ko00310
90	Pantothenate and CoA biosynthesis	72 (0.27%)	ko00770
91	Benzoxazinoid biosynthesis	70 (0.26%)	ko00402
92	Sulfur metabolism	65 (0.24%)	ko00920
93	Glycosaminoglycan degradation	63 (0.24%)	ko00531
94	Isoflavonoid biosynthesis	62 (0.23%)	ko00943
95	Tropane, piperidine and pyridine alkaloid biosynthesis	61 (0.23%)	ko00960
96	Circadian rhythm-mammal	59 (0.22%)	ko04710
97	Linoleic acid metabolism	56 (0.21%)	ko00591
98	Butanoate metabolism	56 (0.21%)	ko00650
99	Fatty acid biosynthesis	55 (0.21%)	ko00061
100	Fatty acid elongation	49 (0.18%)	ko00062
101	Glycosphingolipid biosynthesis—ganglio series	44 (0.16%)	ko00604
102	Riboflavin metabolism	44 (0.16%)	ko00740
103	Folate biosynthesis	43 (0.16%)	ko00790
104	Lysine biosynthesis	40 (0.15%)	ko00300

Table S1. *Cont.*

Number	Pathway	All Genes with Pathway Annotation (26776)	Pathway ID
105	Valine, leucine and isoleucine biosynthesis	40 (0.15%)	ko00290
106	Vitamin B6 metabolism	40 (0.15%)	ko00750
107	Nicotinate and nicotinamide metabolism	38 (0.14%)	ko00760
108	Non-homologous end-joining	37 (0.14%)	ko03450
109	Selenocompound metabolism	36 (0.13%)	ko00450
110	Monoterpenoid biosynthesis	36 (0.13%)	ko00902
111	One carbon pool by folate	35 (0.13%)	ko00670
112	Photosynthesis-antenna proteins	33 (0.12%)	ko00196
113	Arachidonic acid metabolism	33 (0.12%)	ko00590
114	Histidine metabolism	32 (0.12%)	ko00340
115	Glucosinolate biosynthesis	32 (0.12%)	ko00966
116	Other types of *O*-glycan biosynthesis	30 (0.11%)	ko00514
117	Glycosphingolipid biosynthesis—globo series	29 (0.11%)	ko00603
118	Thiamine metabolism	26 (0.1%)	ko00730
119	Indole alkaloid biosynthesis	25 (0.09%)	ko00901
120	Sulfur relay system	22 (0.08%)	ko04122
121	Taurine and hypotaurine metabolism	18 (0.07%)	ko00430
122	Anthocyanin biosynthesis	16 (0.06%)	ko00942
123	Synthesis and degradation of ketone bodies	11 (0.04%)	ko00072
124	C5-Branched dibasic acid metabolism	10 (0.04%)	ko00660
125	Lipoic acid metabolism	8 (0.03%)	ko00785
126	Biotin metabolism	8 (0.03%)	ko00780
127	Caffeine metabolism	4 (0.01%)	ko00232
128	Betalain biosynthesis	3 (0.01%)	ko00965

Table S2. Strawberry contigs and unigenes that were homologous with heat shock transcription factor-like genes.

Table S3. Characteristics of genes encoding heat shock transcription factor proteins in *F.* × *ananassa* Duch. cv. *Toyonoka*.

Table S4. Primers designed for RT-PCR analysis in this study.

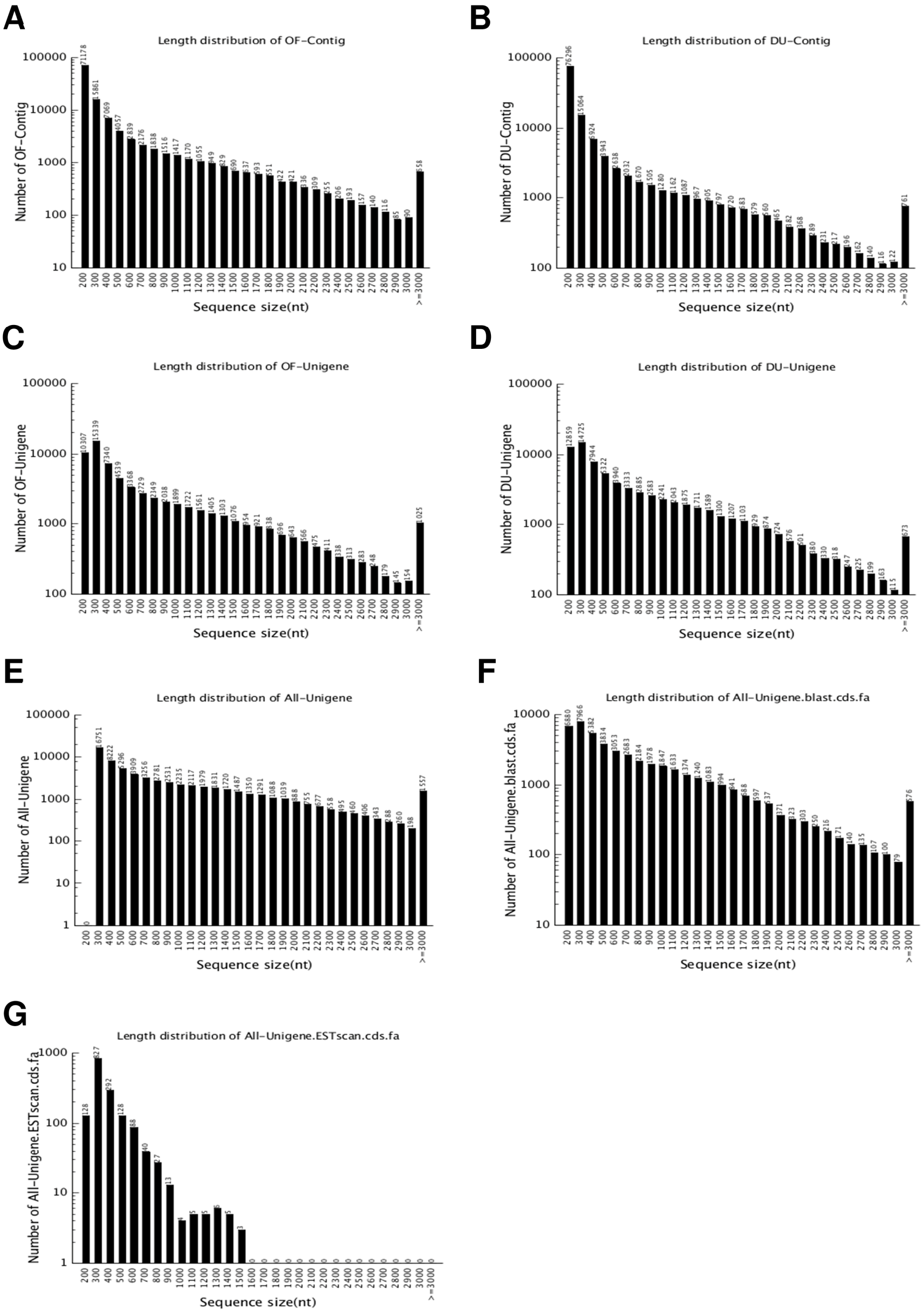

Figure S1. Overview of the size distribution of contigs, unigenes, CDS, and ESTs from 3 strawberry transcriptome assemblies. (**A**) The size distribution of the OF-contigs obtained from our de novo assembly of high-quality clean reads generated from OF transcriptome database; (**B**) The size distribution of the DU-contigs obtained from our de novo assembly of high-quality clean reads established from the DU transcriptome database; (**C**) The size distribution of the OF-unigenes produced from further assembly of OF-contigs; (**D**) The size distribution of the DU-unigenes developed from further assembly of DU-contigs; (**E**) The size distribution of the ALL-unigenes created by combining OF and DU-unigenes and removing the overlapped unigenes from these 2 databases; (**F**) The size distribution of the CDS produced by searching ALL-unigene sequences against Nr, SWISS-PROT, KEGG, and COG databases, in that order, by using the BLASTX program (*E*-value < 10^{-5}); (**G**) The size distributions of the ALL-unigene ESTs retrieved from the ESTScan results. For ALL-unigene CDS that had no hits in the searched databases, the BLAST results were subjected to ESTScans and subsequently translated into peptide sequences.

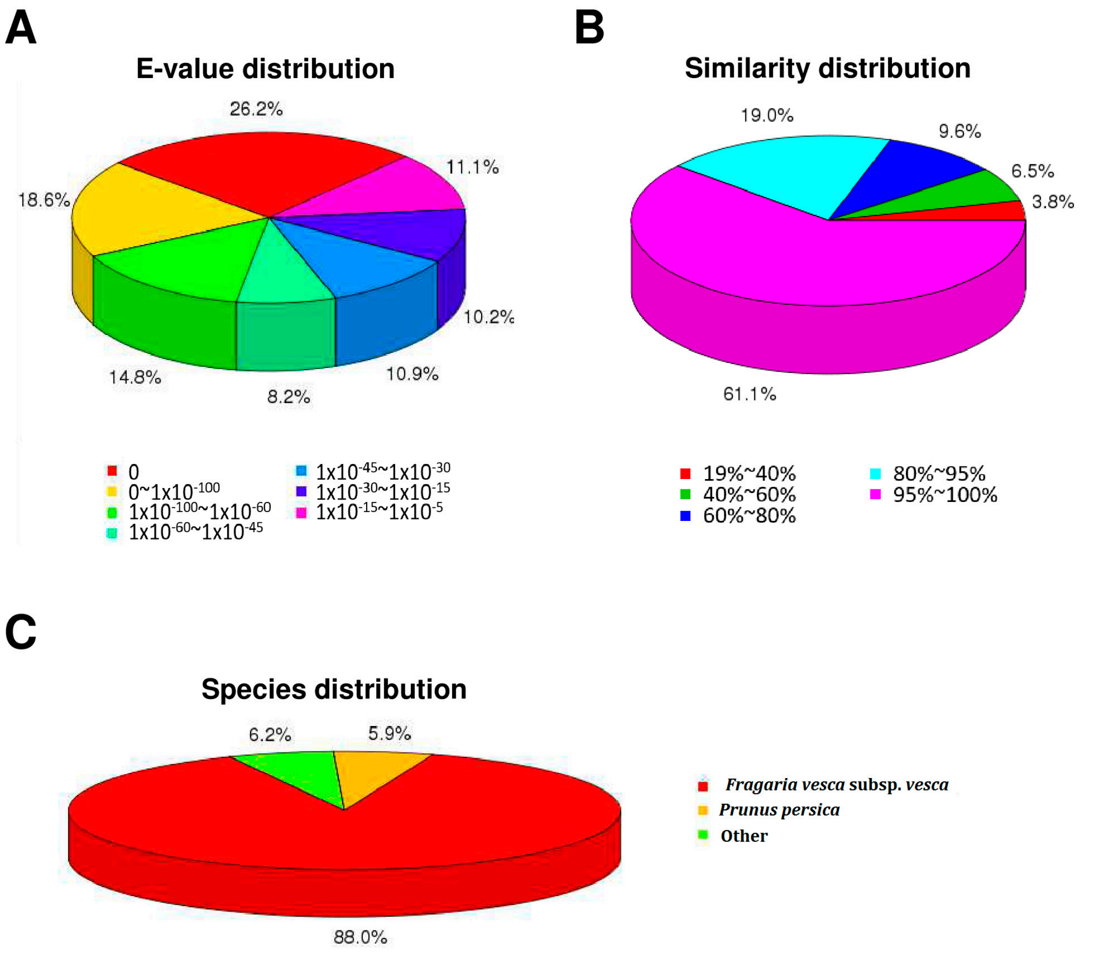

Figure S2. Characteristics of the homology search of the assembled sequences against the Nr database. (**A**) The *E*-value distribution of BLAST hits for each unique sequence, with a cut-off *E*-value of $\leq 10^{-5}$; (**B**) Similarity distribution of the top BLAST hits for each sequence; (**C**) Species distribution is shown as a percentage of the total homologous sequences with *E*-value $\leq 10^{-5}$. The first hit of each sequence was used for statistical analysis.

A

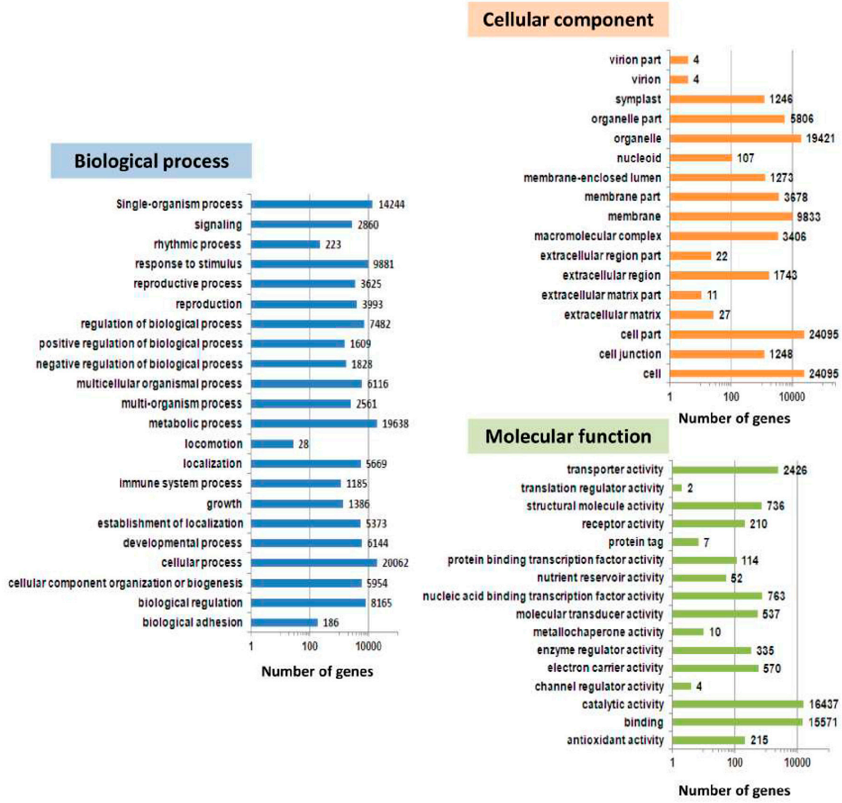

B

COG categories	Functional classification	Gene-Number
A	RNA processing and modification	266
B	Chromatin structure and dynamics	330
C	Energy production and conversion	929
D	Cell cycle control, cell division, chromosome partitioning	2231
E	Amino acid transport and metabolism	1338
F	Nucleotide transport and metabolism	321
G	Carbohydrate transport and metabolism	2294
H	Coenzyme transport and metabolism	598
I	Lipid transport and metabolism	890
J	Translation, ribosomal structure and biogenesis	2296
K	Transcription	4439
L	Replication, recombination and repair	3437
M	Cell wall/membrane/envelope biogenesis	2033
N	Cell motility	410
O	Posttranslational modification, protein turnover, chaperones	2982
P	Inorganic ion transport and metabolism	1031
Q	Secondary metabolites biosynthesis, transport and catabolism	1167
R	General function prediction only	6259
S	Function unknown	2609
T	Signal transduction mechanisms	2889
U	Intracellular trafficking, secretion, and vesicular transport	1155
V	Defense mechanisms	485
W	Extracellular structures	17
Y	Nuclear structure	6
Z	Cytoskeleton	590

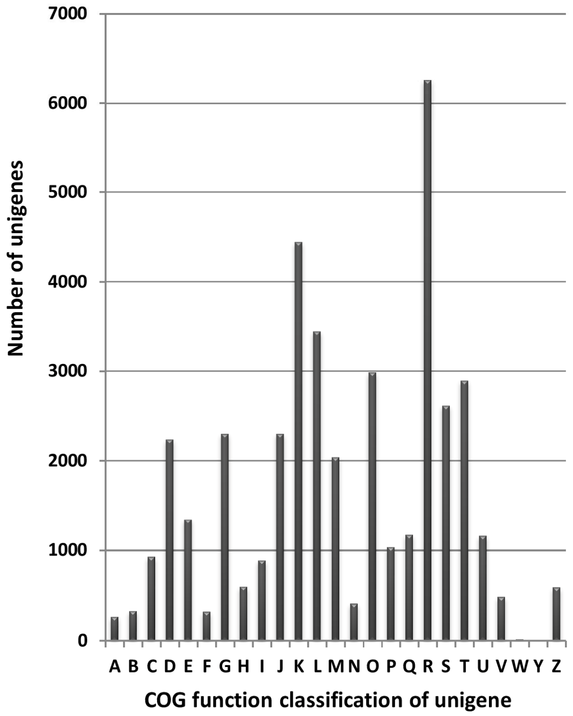

Figure S3. GO and COG classification of the strawberry transcriptome. (**A**) The GO classification of the strawberry transcriptome. Annotated sequences were classified into Biological Process, Cellular Component, and Molecular Function groups in addition to 55 sub-categories (functional groups); (**B**) The COG functional classification of all unigene sequences. Out of 47,794 Nr hits, 17,441 sequences were assigned to 25 separate COG classifications.

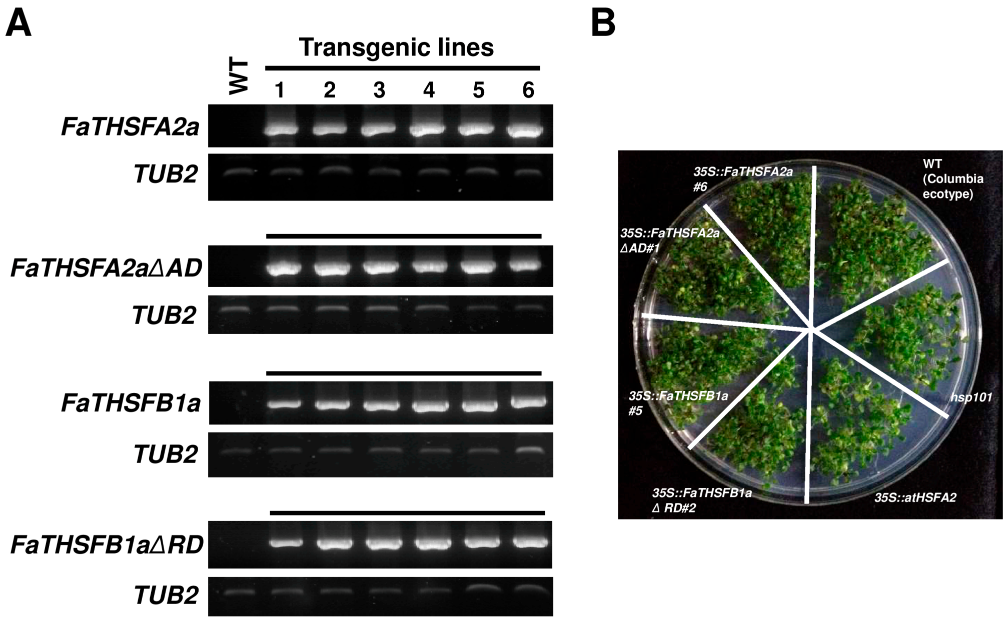

Figure S4. Generation of *FaTHSFA2a* and *FaTHSFB1a*-overexpressing transgenic *Arabidopsis* plants. (**A**) The electrophoresed gel displayed high expression of *FaTHSFA2a, FaTHSFA2aΔAD, FaTHSFB1a,* and *FaTHSFB1aΔRD* in transgenic lines #1–#6. Total RNAs extracted from 10-day-old plants grown in MS medium at 22 °C were used in RT-PCR analysis with gene-specific primer sets (Table S4). *TUB2* was used as the loading control; (**B**) The photographs of 12-day-old transgenic lines (*FaTHSFA2a#6, FaTHSFA2aΔAD#1, FaTHSFB1a#5, FaTHSFB1aΔRD#2,* and *35S::atHSFA2*), *hsp101* mutant, and wild-type (WT) were cultivated on 1/2MS media at 22 °C. Transgenic lines are indistinguishable from WT *Arabidopsis* considering the phenotype during growth under normal conditions.
